# Unveiling the Role of PNMA2 in Endometriosis: From Proliferation and Apoptosis to Immunomodulation

**DOI:** 10.1111/jcmm.70576

**Published:** 2025-05-05

**Authors:** Mengjun Zhang, Zidi Zhang, Jialin Wang, Haodi Yue, Xueling Lou, Quanling Feng, Lindong Zhang

**Affiliations:** ^1^ Department of Gynecology The Third Affiliated Hospital of Zhengzhou University Zhengzhou China; ^2^ Department of Orthopedics The First Affiliated Hospital of Zhengzhou University Zhengzhou China; ^3^ Department of Center for Clinical Single Cell Biomedicine Henan Provincial People's Hospital, People's Hospital of Zhengzhou University Zhengzhou China

**Keywords:** apoptosis, endometriosis, immunomodulation, machine learning, PNMA2

## Abstract

Endometriosis is a chronic disease that jeopardises the quality of life of about 10% of women. The aim of this study was to investigate the expression, function, regulatory mechanism, and relationship with immune cell infiltration of PNMA2 in endometriosis. This study investigates the potential involvement and regulatory mechanisms of PNMA2 in the development of endometriosis through the integration of public data, machine learning, clinical sample transcriptome sequencing, and in vitro cell experiments. Cytological in vitro experiments were conducted to validate the impact of PNMA2 on the modulation of proliferation, migration, apoptosis, and autophagy in 12z cells. Rescue experiments were performed based on the autophagy activator (RAPA) to clarify the regulatory details of PNMA2, apoptosis, and autophagy. The Ciberort algorithm was employed to discern the association between PNMA2 gene expression and more than 20 distinct immune cell infiltration types in endometriosis. The expression of PNMA2 exhibited a notable increase in individuals with endometriosis. Knockdown of PNMA2 inhibited the proliferation and migration of 12z cells. Knockdown of PNMA2 could directly promote apoptosis and could also inhibit autophagy and indirectly promote apoptosis. PNMA2 displayed associations with immune cell infiltration and immunomodulation. The present study demonstrated that the up‐regulation of PNMA2 was associated with malignant growth, anti‐apoptosis, and immunoregulation of human endometriotic cells. Therefore, PNMA2 may serve as a new diagnostic biomarker and a promising therapeutic target.

**Abbreviations**
EMs: endometriosis
GEO: Gene Expression Omnibus
GO: Gene Ontology
KEGG: Kyoto Encyclopedia of Genes and Genomes
LASSO: Least Absolute Shrinkage and Selection Operator
PNMA2: PNMA Family Member 2
ROC: receiver operating characteristic
SVM: support vector machine

## 1 Introduction

Endometriosis (EMs) is a non‐malignant neoplasm distinguished by the existence of functional endometrial tissue beyond the confines of the uterine cavity, exhibiting a benign histopathological pattern but displaying malignant clinical characteristics such as invasion, metastasis, and recurrence [1]. Furthermore, endometriosis is acknowledged as a precancerous ailment, with approximately 2.5% of women afflicted by endometriosis eventually developing endometriosis‐associated ovarian cancer [2]. The proliferation of tumour cells is influenced by genetically inherited factors, leading to the systematic production of substantial quantities of hormones, growth factors, and cytokines that contribute to the progression of the disease. However, there is still a lack of comprehensive understanding of whether endometriosis cells, which are similar to tumour cells, can cause changes in the genetic regulatory microenvironment that lead to endometriosis progression [3]. Related research posits that the active involvement of inflammatory factors and immune cell infiltration in the peritoneal fluid of endometriosis patients plays a significant role in the development and advancement of endometriosis. Nevertheless, further investigation is required to elucidate the influencing factors and molecular mechanisms involved in this process [4, 5, 6].

PNMA2 belongs to the paraneoplastic Ma antigen (PNMA) family, which is associated with paraneoplastic disease (PND) [7, 8, 9, 10]. The PNMA gene family has been identified as the primary causative driver gene in several highly aggressive forms of uterine cancer observed in clinical settings. This gene family is responsible for mediating the abnormal cellular signalling pathways associated with apoptosis and various types of cancer [11]. Notably, the aberrant expression profiles of the cellular signalling regulator PNMA2 indicate its potential involvement in tumorigenesis. Consequently, its expression profile is frequently utilised as a molecular diagnostic tool, highlighting its significance in the field of cancer research. The molecular diagnostic significance and mechanism of action of PNMA2 in endometriosis remain unclear. Among the members of the PNMA family, MOAP‐1 has been extensively investigated and is known to facilitate apoptotic signalling through its interaction with Bax, a pro‐apoptotic member of the Bcl‐2 family [12, 13, 14]. This observation has prompted us to investigate the potential association between PNMA2 and the regulation of apoptosis in endometriosis. Excitingly, the present manuscript experimentally validates the malignant biological behaviour and apoptosis‐associated signal pathways of PNMA2 in endometriotic cells by in vitro cellular experiments.

Numerous studies conducted thus far have demonstrated that immune irregularities potentially exert a significant influence on the progression of endometriosis. For instance, the presence of immune cells, such as macrophages and natural killer (NK) cells, within the peritoneal cavity of individuals with endometriosis disrupts the local peritoneal microenvironment, thereby facilitating the metamorphosis and proliferation of ectopic endometrial cells [15, 16, 17]. Given the intricate and multifaceted nature of endometriosis pathogenesis, it is postulated that aberrant gene expression impacting apoptosis, in conjunction with modifications in the immune microenvironment, synergistically contributes to the initiation and advancement of endometriosis.

In this investigation, we employed the GEO dataset to ascertain target genes and examined their impact on cellular malignant biological behaviours (apoptosis, autophagy and immunomodulation) via validation of machine learning, clinical sample transcriptome sequencing and cellular experimentation. Following this, we conducted an analysis on the infiltration patterns of 22 distinct subtypes of immune cells in both endometriosis and normal tissues, while also examining the relationship between PNMA2 and immune cells. Given the dearth of reliable diagnostic markers and therapeutic targets for endometriosis, our goals were to determine whether PNMA2 could serve as an early non‐invasive diagnostic target for endometriosis and to explore it as a potentially effective therapeutic target for the disease.

## 2 Materials and Methods

### 2.1 Data Collection

The Gene Expression Overview database (GEO database, http://www.ncbi.nlm.nih.gov/geo/) is a widely recognised and extensive database platform. From this platform, we obtained endometriosis‐related datasets (GSE7305, GSE23339, GSE25628, GSE51981, and GSE58178). After conducting a search and applying filters, we selected two datasets that contained gene expression data (GSE51981, *n* = 148; GSE7305, *n* = 20). GSE7305 was chosen as the verification. To ensure comparability, the combined datasets underwent batch normalisation using the R packages “SVA” and “LIMMA”. The data were sourced from the GPL570 platform (Affymetrix Human Genome U133 Plus 2.0 array). The dataset GSE51981 comprises mRNA data obtained from the endometrium of 77 patients diagnosed with endometriosis and 71 healthy women. Similarly, the dataset GSE7305 includes mRNA information from the endometrium of 10 patients with endometriosis and 10 healthy women. The extraction of gene expression matrices was performed using Perl scripts, and the annotation of probes was conducted based on the platform annotation file. Turku Endometriosis Database (Endomet, https://endometdb.utu.fi/) was used to explore the correlation between PNMA2 and clinical features of endometriosis.

### 2.2 Data Preprocessing and Analysis

Identification of differentially expressed genes in endometriosis: We downloaded normalised and log‐transformed GSE51981 data and screened for DEGs with a log value of 0.5 and a *p*‐value of < 0.05 using combined and batch‐normalised gene expression data transformed by Log2. Heatmaps and volcano maps of differential genes were plotted using R software (Version 4.0.2, Robert Gentleman and Ross Ihaka, New Zealand). Redundant genes were eliminated from 28 gene regulators using 10‐fold LASSO regression, resulting in the identification of endometriosis‐related core regulators. Machine learning models were constructed, including random forest (RF), support vector machine (SVM) and LASSO regression. The area under the ROC curve (AUC) values of the different models were compared. The findings are depicted in a Venn diagram. The functional enrichment analyses conducted in this study encompassed Gene Ontology (GO)/Kyoto Encyclopedia of Genes and Genomes (KEGG) analysis, as well as Gene Set Enrichment Analysis (GSEA). The samples were stratified into PNMA2 high and low expression groups, utilising the median PNMA2 value as the threshold, and differential genes for GO/KEGG analysis were identified using the LIMMA packages. In this study, we applied Ciberort, an excellent tool for calculating the abundance of specific cells in a mixing matrix, to identify immune cell subpopulations in GSE51981. Algorithms were used to distinguish the relative proportions and correlations of PNMA2 gene expression with 22 different types of immune‐permeable cells in women with and without endometriosis. Spearman correlation analysis was used, and statistically significant values were defined as *p* < 0.05.

### 2.3 Patient Data and Clinical Tissue Samples

RNA‐seq analysis was performed on paired tissue samples of in situ and ectopic endometrial tissue from 10 patients who underwent laparoscopic surgery in 2022–2023 at the Third Affiliated Hospital of Zhengzhou University, as well as 5 controls of normal endometrium who underwent surgery due to other benign gynaecological diseases. The patients signed informed consent and complied with the ethical review of the Third Affiliated Hospital of Zhengzhou University (No. 2023‐098). All endometriosis patients were staged according to the American Fertility Society's revised endometriosis staging criteria (AFS‐R). All collected endometrial tissues were diagnosed as proliferative endometrium by pathohistology. There was no statistically significant difference in the age of the patients in each group (*p* > 0.05). All patients had normal menstrual cycles, were not pregnant or breastfeeding, had not been taking hormones for 6 months prior to surgery, and no significant medical or surgical diseases or complications were found.

### 2.4 RNA Extraction and Library Construction

Total RNA was extracted using TRIzol reagent according to the instructions. Transcriptome sequencing and analysis were performed by Shanghai Ouyi Biotechnology Co. Genes (counts) were subjected to PCA analysis and mapping using R (v 3.2.0) to assess sample biological replicates. Differentially expressed genes (DEGs) were analysed using DESeq2 software, where genes that met the threshold of *q* value < 0.05 and fold change > 2 or fold change < 0.5 were defined as differentially expressed genes (DEGs). Subsequently, GO, KEGG Pathway and GSEA enrichment analyses of differentially expressed genes were performed based on the hypergeometric distribution algorithm, which was used to screen for significantly enriched functional entries. R (v 3.2.0) was used to plot bar charts, chord charts or enrichment analysis circle plots for significantly enriched functional entries.

### 2.5 Cell Culture and Cell Transfection

Human endometriosis 12z cells and normal endometrial cells hEEC were inoculated in 6‐well plates at 0.8 × 10^5^ cells/well and incubated at 37°C for 24 h. A serum‐free optimal basal medium was made by mixing 20 nM of small interfering ribonucleic acid with 5 μ liposome L reagent (Invitgen, CALS‐BAD, CA, CA, OPTI‐MEM). Cells were taken 24 h after transfection and used for subsequent experiments. The siRNAs for PNMA2 were si‐1078, si‐1376, and si‐1638. The siRNA sequence (si‐1078) was sense(5′‐CUGCAUCUCACCAGAAUUATT‐3′) and antisense(5′‐UAAUUCUGGUGAGAUGCAGTT‐3′). The siRNA sequence (si‐1376) was sense(5′‐GAGGCCUUUAAGCAAGUGUTT‐3′) and antisense(5′‐ACACUUGCUUAAAGGCCUCTT‐3′). The siRNA sequence (si‐1638) was sense(5′‐CCAGCUUCCUUGAGCUAAUTT‐3′) and antisense(5′‐AUUAGCUCAAGGAAGCUGGTT‐3′). After gene knockdown and cell transfection, the expression level of PNMA2 was explored by qRT‐PCR and Western blotting, and then the appropriate siRNA was selected for a series of subsequent cell experiments.

### 2.6 Ki‐67 Cell Immunofluorescence

Cells are added uniformly to a 24‐well plate and fixed with 4% paraformaldehyde when the cell density reaches 50%–60%. Use 0.5% Triton X‐100 for 20 min. After rinsing with PBS, block with 10% goat serum for 1.5 h at 37°C. Add primary antibody (Ki‐67, 1:200) to each well and incubate overnight. Add secondary antibody and incubate at 37°C for 1 h in the dark. Finally, stain the nuclei with DAPI. Under fluorescence observation using a fluorescence microscope, images were acquired randomly from five fields of view. The number of surviving and proliferating cells was measured and recorded using ImageJ software.

### 2.7 CCK8 Assay

The cell suspension was homogeneously added to 96‐well plates with 100 μL per well. 10 μL of CCK‐8 reagent (Yersen, Shanghai, China) was added to 90 μL of culture medium after cell attachment. After 2 h of incubation, a microtiter plate assay was performed. The detection times were 0, 12, 24, 48, and 72 h. The optical density was measured by OD (wavelength 450 nm) using a microplate reader (Thermo Fisher Scientific).

### 2.8 qRT‐PCR

Total RNA was extracted from cells using Total RNA Kit I (Omega Biotek). RNA reverse transcription was performed under the guidance of NovoScript Plus All‐in‐one 1st Strand cDNA Synthesis SuperMix (RNA reverse transcription was performed under the guidance of NovoScript Plus All‐in‐one 1st Strand cDNA Synthesis SuperMix (Novoprotein). The primer sequences for GAPDH and PNMA2 were as follows (GAPDH‐F: 5′‐CAAGGTCATCCATGACAACTTTG) and GAPDH‐R: 5′‐GTCCACCACCCTGTTGCTGTAG‐3′), (PNMA2‐F: 5′‐CTGGGCAGGTATAGACTGCTT‐3′ and PNMA2‐R: 5′‐TGGCCGAGACATCAGTATCTT‐3′). Thermal cycling conditions were as follows: initial denaturation at 95°C for 10 min, denaturation for 10 s, annealing and extension at 60°C for 30 s for a total of 40 cycles.

### 2.9 Tunel Assay

Cells were fixed in 4% paraformaldehyde for 5 min at 4°C, then washed with PBS. Cells were blocked with 5% skimmed milk and 0.1% Triton X‐100 for 1 h at room temperature, then incubated with tunel primary antibody (1:500, Sigma Aldrich, St. Louis, MO, USA) for 1 h at 4°C, then incubated with secondary antibody (1:500, Sigma Aldrich, St. Louis, MO, USA) for 1 h. The cells were then washed with PBS buffer. secondary antibody (1:500, Sigma Aldrich, St. Louis, MO, USA) for 1 h at 4°C, and then washed with PBS buffer. Nuclei were identified using DAPI. Stained cells were observed with a fluorescence microscope (IX73, Olympus, Tokyo, Japan) in five non‐overlapping fields of view.

### 2.10 Western Blotting

Total protein was extracted using a protein extraction kit. Protein concentration was detected using a BCA kit. Proteins were separated by SDS‐PAGE and transferred to a PVDF membrane. Skimmed milk was closed and incubated overnight with primary antibody in Table S1. membranes were washed with TBST and incubated with secondary antibody and visualised with CEL. For clinically collected tissue samples, the same tissue was repeated three times and statistically analysed for relevant protein content detection based on Western blotting. Finally, all the results of all tissues were merged and statistically analysed. Rescue experiments were performed based on the autophagy activator (RAPA) and Western blotting to clarify the regulatory details of PNMA2, apoptosis, and autophagy.

### 2.11 Clonogenic Assay

Spread the transfected cell suspension evenly on a 6‐well plate with 1000 cells per well. Replace the medium with fresh medium on day 4 and observe the formation of cell colonies after 10 days. The cells were fixed with 4% paraformaldehyde for 30 min and stained with 0.1% crystal violet for 5 min. The number of colonies formed was measured and counted using ImageJ (version 1.52).

### 2.12 Wound‐Healing Assay

Inoculate transfected 12z cells in 6‐well plates. Remove detached cells by drawing a straight line across each well with a 200 μL sterile pipette tip and rinsing three times with PBS. The cells were added with fresh serum‐free medium. Scratches at the same site were photographed with a microscope at 0 h and 24 h, respectively.

### 2.13 Transwell Assay

100 μL of transfected 12z cell suspension and control cell suspension were seeded into the upper chamber of transwell plates (Corning Costar, Shanghai, China). 600 μL of essential medium with 20% FBS was added to the lower chamber. After 24 h, 4% paraformaldehyde was fixed for 15 min. Stain with 0.1% crystal violet for 5 min. The upper chamber is then wiped with a cotton swab and the cells are viewed microscopically and photographed. Measure and record the number of migrating cells using ImageJ (version 1.52).

### 2.14 Flow Cytometry

Digest, centrifuge, and resuspend transfected cells. Add pre‐cooled 75% ethanol to the cell suspension The tubes containing the cell suspension were then left at −20°C overnight. Remove RNA contaminants from the cell suspension. 10 μL of cell suspension was added to 5 μL of propidium iodide (PI) and 5 μL of FITC‐Annexin V and stained for 20 min. The distribution of apoptotic cells was detected by flow cytometry (Canto plus, BD, USA).

### 2.15 Statistical Analysis

Data were analysed using SPSS 25.0 software and R software (Version 4.0.2); continuous variables were described by mean ± standard deviation, and *t*‐tests were used to compare whether the data were normally distributed or not. Paired × tests were used for count data, and logistic regression models were used for significance testing of relevant clinical indicators. All statistical tests were considered statistically significant when the *p*‐value was less than 0.05.

## 3 Results

### 3.1 Machine Learning‐Based Screening and Identification of Key Genes for Endometriosis

**FIGURE 1:**
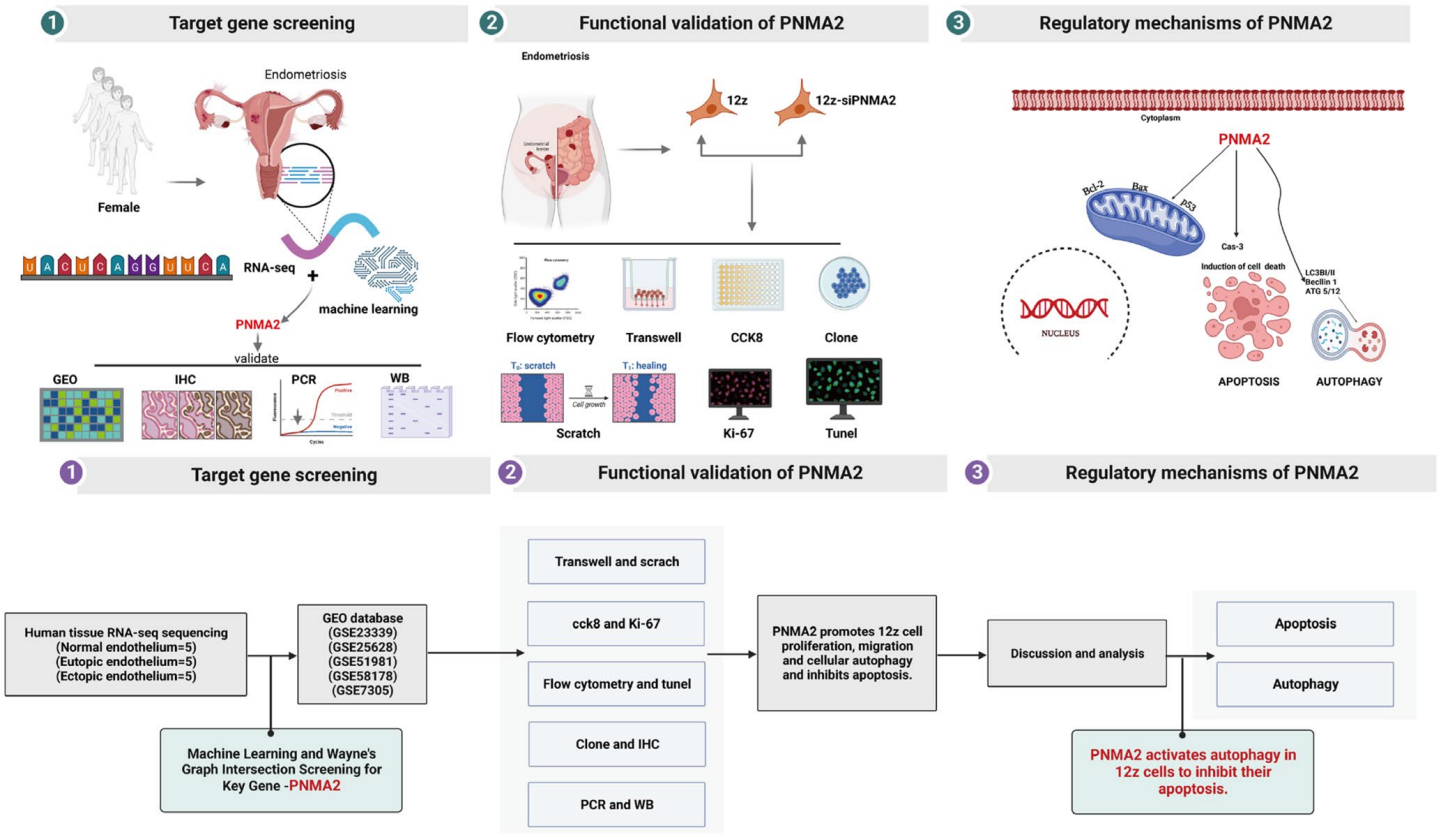
Overview of the study workflow.

**FIGURE 2:**
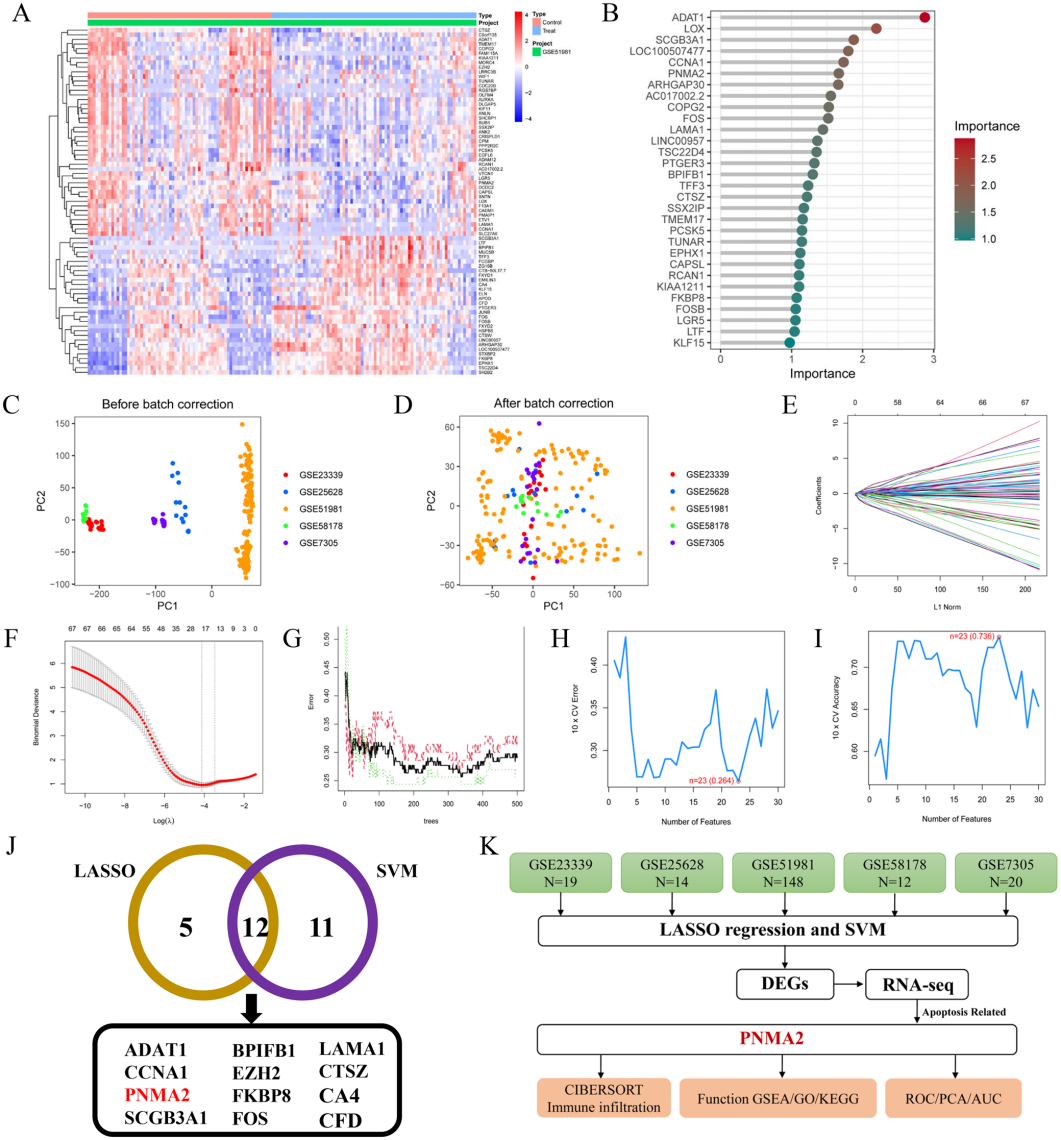
Bioinformatics screening and machine learning of key genes for endometriosis. (A) The expression of DEGs was visualised by heatmap. (B) Lollipop plot of Random Forest Tree screening for disease signature genes. (C, D) Composition distribution map of GEO data after preprocessing using PCA analysis. (Red spots: GSE23339, blue spots: GSE25628, orange spots: GSE51981, green spots: GSE58178, purple spots: GSE7305) Horizontal coordinate PC1 (Principal Component 1) represents the direction of the data with the highest variability; vertical coordinate PC2 (Principal Component 2) represents the direction of the data with the second highest variability. (E, F) Lasso‐regression screening for disease‐characterised genes. Least absolute shrinkage and selection operator (LASSO) algorithm shows the optimal coefficient and minimal lambda of the DE‐MRGs. (G) Randomised forest tree screening for disease signature genes. (H, I) SVM machine learning screening for disease signature genes. (J) Wayne diagram of Lasso regression and SVM machine learning screening for disease signature genes. Key genes were screened (ADAT1, CCNA1, PNMA2, SCGB3A1, LAMA1, CTSZ, BPIFB1, EZH2, FBP8, FOS, CA4, CFD). (K) Bioinformatics screening and machine learning flowchart for key endometriosis genes.

The overall experimental flow of this study is shown schematically in Figure 1. We obtained datasets related to endometriosis (GSE51981 = 148 and GSE7305 = 20) from the GEO platform. Seventy‐one normal endometrial samples and 77 endometriosis samples in the GSE51981 dataset were analysed to obtain differentially expressed genes, and the heatmap of gene expression of up‐ and down‐regulated Top75 is shown in Figure 2A. Following this, the Top30 genes were additionally screened using the correlation index (Figure 2B) to further assess their value as core genes. Diagnostic biomarkers of interest were identified through the utilisation of two distinct algorithms. Subsequently, a sample batch evaluation of the dataset was conducted, and the LASSO logistic regression algorithm was employed to determine the feature variables associated with EMS from deg. (Figure 2C–F). The SVM algorithm was used to identify 23 feature subsets in the determined deg. (Figure 2G–I). Based on the above two machine learning algorithms of LASSO and SVM, 12 core genes (ADAT1, CCNA1, PNMA2, SCGB3A1, BPIFB1, EZH2, FKBP8, FOS, LAMA1, CTSZ, CA4 and CFD) were screened out, as shown in Figure 2J. By reviewing relevant studies, we targeted the PNMA2 gene, which has not been reported in endometriosis diseases. The flowchart of machine learning screening of core genes is shown in Figure 2K.

### 3.2 Expression and Functional Validation of PNMA2 in Endometriosis Tissues

**FIGURE 3:**
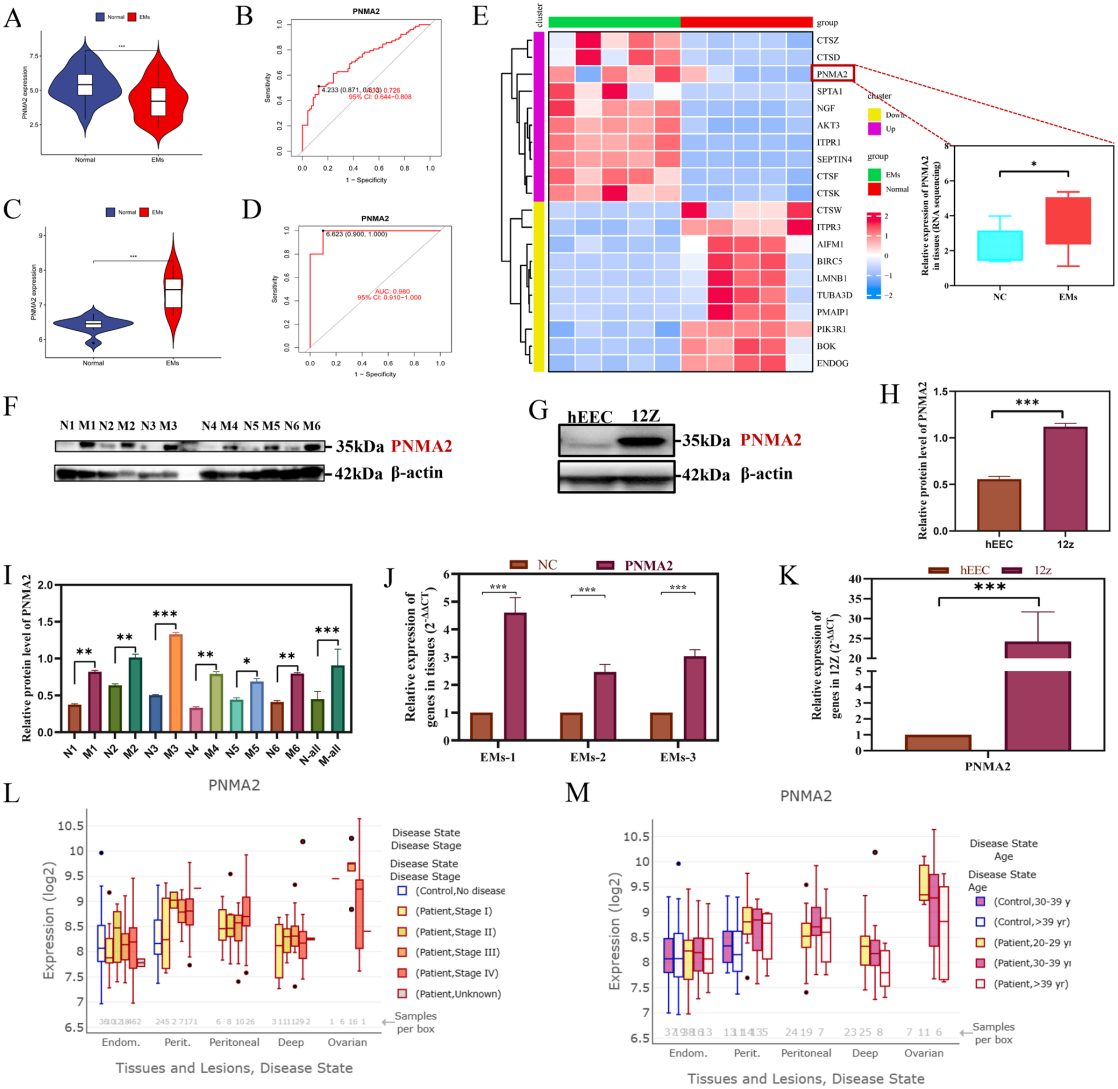
PNMA2 differential expression analysis, validation and Correlation of clinical characteristics. (A) Differential expression violin plots of PNMA2 gene in the endometriosis group and normal control group in datasets (GSE51981, normal = 71, endometriosis = 77) in the GEO database. (B) Dataset GSE51981 of PNMA2 gene in endometriosis diagnosis ROC curves. (C) Differential expression violin plots of PNMA2 gene in the endometriosis group and normal control group validated by dataset GSE7305 in the GEO database. (D) Dataset GSE7305 validation of PNMA2 gene in endometriosis diagnosis ROC curves. (E) Differential expression heatmap of 5 pairs of clinical samples transcriptome sequencing data validating PNMA2 in the endometriosis group and normal control group. Heatmap of apoptosis‐related TOP20 genes. Box plot of differential expression of the PNMA2 gene, blue represents ectopic endothelial tissue and red represents eutopic endometrium of endometriosis. (F) Protein immunoblot expression of PNMA2 in 6 pairs of clinical tissue specimens from the endometriosis group and normal control group. (G) Protein immunoblot expression of PNMA2 in normal endometrial cells (heec) and endometriotic cells (12z). (H) Immunoblotting statistical histograms of normal endometrial cells (heec) and endometriotic cells (12z). (I) Western blot statistical histogram of clinical tissues of endometriosis and normal control endometrium (comparison between each subgroup and the total group). (J) PCR graph of 4 pairs of clinical tissue specimens from the endometriosis group and normal control group. (K) PCR graph of expression of PNMA2 in normal endometrial cells (heec) and endometriotic cells (12z). (L) Box plot of correlation between PNMA2 gene expression and clinical stage of the disease based on the Endomet database (Turku Endometriosis Database). (M) Box plot of correlation between PNMA2 gene expression and age of the disease based on the Endomet database (Turku Endometriosis Database).

Utilising machine learning techniques, the present study conducted a comprehensive analysis of the core gene PNMA2, whereby its clinical diagnostic value was initially assessed through the evaluation of its ROC and AUC values (Figure 3A,B). Subsequently, the GSE7305 dataset was employed to validate the expression and ROC diagnostic value of the aforementioned core gene. Notably, Figure 3C,D demonstrated a significant upregulation of PNMA2 in endometriosis tissues, with an exceptionally high AUC value of 0.98. In contrast, the previous study utilising GSE51981 exhibited an inverse expression pattern. Considering that this discrepancy may be due to statistical bias caused by dataset batch differences and sample size limitations, transcriptome sequencing was performed on clinical sample tissues (*n* = 15 cases) to further explore and verify the expression level of PNMA2. The analysis revealed that PNMA2, a gene associated with apoptosis, exhibited significantly elevated expression levels in endometriosis tissues, as depicted in Figure 3E Heatmap. This finding suggests that PNMA2 may potentially serve as a pathogenic gene in endometriosis. Next, based on clinical collections of patient endometriosis tissue (M1‐6) and normal control endometrium (N1‐6), our analysis revealed a significant upregulation of PNMA2 in both endometriosis tissues and cell lines, as compared to their normal counterparts, as indicated by the mRNA and proteomic expression profiles of human tissues and cell lines (Figure 3F–K). Clinicians displayed an exceptional level of concern regarding the potential association between differentially expressed target genes and clinical features. Subsequent analysis of the Endomet database unveiled that the atypically elevated expression of PNMA2 in individuals afflicted with endometriosis exhibited a correlation with both the clinical stage and age of the disease. Notably, the expression level of PNMA2 tended to be higher in stages II‐IV compared to stage I across various forms of endometriosis. Furthermore, the findings indicate that patients aged 30–39 years exhibit a higher expression level of PNMA2 compared to those aged > 39 years across various types of endometriosis. This correlation between differential expression and clinical features strongly suggests that PNMA2 may have a pivotal role in the development of endometriosis. Therefore, it is imperative to investigate the function and mechanism of PNMA2 in the context of endometriosis.

### 3.3 Effect of Knockdown of PNMA2 on the Malignant Function of 12z Cells

**FIGURE 4:**
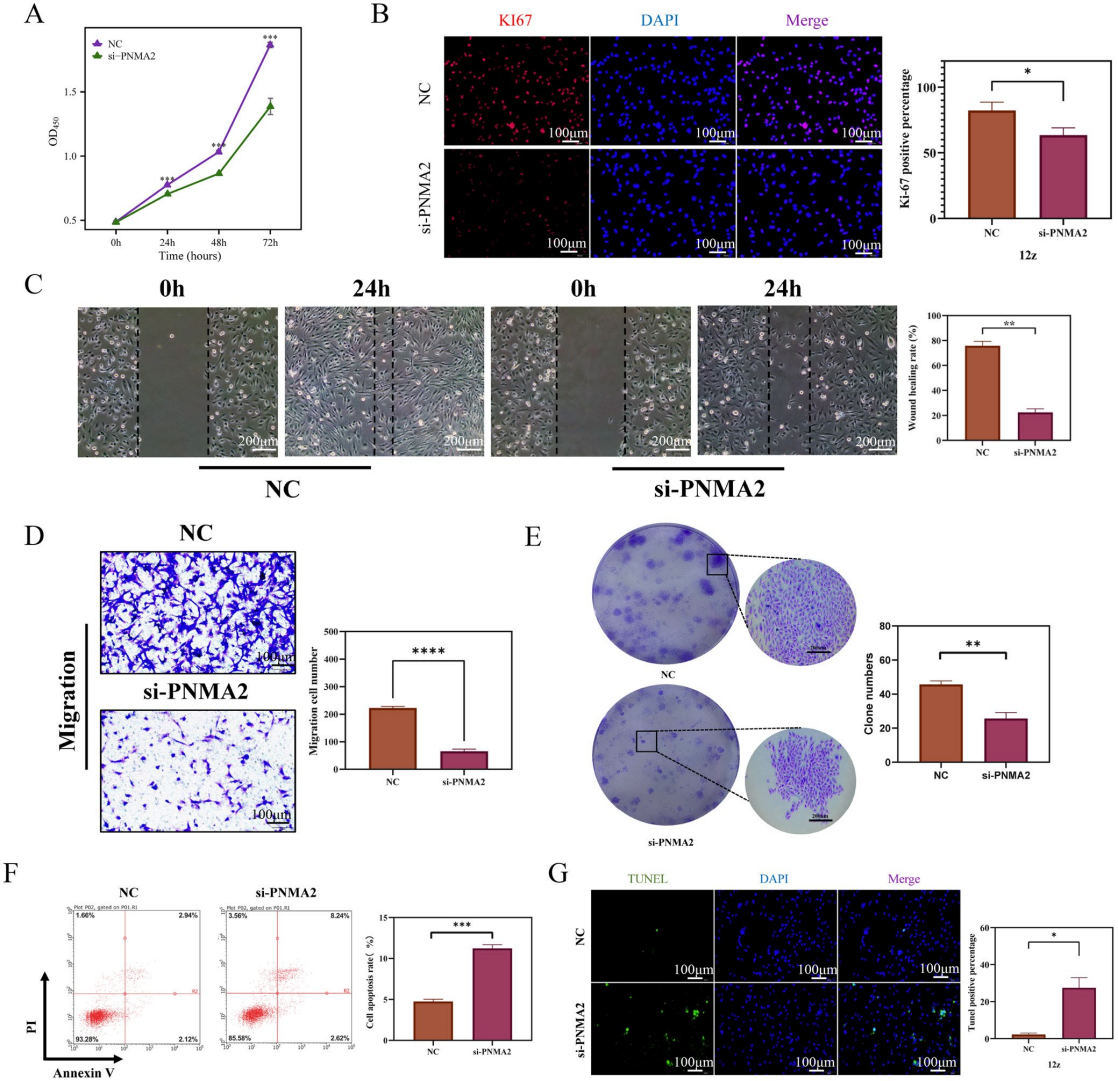
Effects of PNMA2 knockdown on the function of endometriotic cells 12z. (A) Effect of PNMA2 gene knock down on the proliferation of 12z cells. Results of CCK8 experiments after transfection of the 12z cell line. OD values measured at 0 h, 24 h, and 48 h at 450 nm wavelength are shown, and they represent the cell proliferation rate. (B) Ki‐67 immunofluorescence staining of the 12z cell line after transfection. red staining of Ki67 represents cells in a proliferation state. blue nuclear staining of DAPI represents all living cells. (C) Results and statistics of the wound healing assay on 12z cells after PNMA2 gene knock down. The wound healing rate at 0 h and 24 h was measured after transfection. (D) Transwell assay results and statistics of 12z cells after transfection. The number of migrating cells was measured at 24 h after cell transfection. (E) Results and statistics of clone formation capability on 12z cells after PNMA2 gene knock down. (F) Results and statistics of flow apoptosis assay on 12z cells after PNMA2 gene knock down. (G) Tunel immunofluorescence staining of the 12z cell line after transfection. Green staining of tunel represents cells in an apoptosis state. *p ≤ 0.05, **p ≤ 0.01, ***p ≤ 0.001, and ****p ≤ 0.0001.

Further investigation revealed that PNMA2 could potentially serve as a biomarker that impacts the development of endometriosis. Consequently, we conducted plasmid transfection of the PNMA2 gene in immortalised epithelial cells (12z) associated with endometriosis in order to disturb the expression of PNMA2. The effectiveness of the knockdown was confirmed through Western blotting (Figure S1A,B) and PCR (Figure S1C). The knockdown with the highest efficiency was chosen for subsequent experiments. In terms of validating cell proliferation, we employed the CCK8 assay and Ki67 immunofluorescence techniques. Notably, Figure 4A demonstrates that there was no notable disparity in cell proliferation between the si‐PNMA2 group and the NC group at 0 h. However, at 24, 48, and 72 h, the cell proliferation in the si‐PNMA2 group exhibited a statistically significant decrease. Furthermore, Figure 4B illustrates that cells in the si‐PNMA2 group exhibited diminished red fluorescence compared to cells in the NC group. These findings collectively suggest that PNMA2 plays a significant role in promoting the proliferation of 12z cells. The validation of cell migration ability was conducted using both scratch assay and transwell assay. The results depicted in Figure 4C,D indicate that the cells in the NC group exhibited a greater scratch healing area and a higher number of migrated cells to the lower chamber compared to those in the si‐PNMA2 group. This indicates that knocking down the PNMA2 gene inhibited the migration ability of 12z cells. Additionally, the validation of the clone forming ability of the cells revealed that the cells in the NC group, as shown in Figure 4E, displayed a greater number and larger size of clonal colonies in comparison to the cells in the si‐PNMA2 group. The findings suggest that PNMA2 enhances the capacity of 12z cells to form clones. In order to validate the impact of PNMA2 on apoptosis in 12z cells, flow apoptosis cytometry and tunel immunofluorescence staining were employed, as depicted in Figure 4F,G. The experimental results indicate that the si‐PNMA2 group exhibited a higher rate of apoptosis and greater tunel fluorescence staining compared to the NC group. The involvement of PNMA2 in the regulation of apoptosis in 12z cells is evident. Through the aforementioned functional investigation of PNMA2 in endometriosis, a benign tumour, we have discovered a novel biomarker, PNMA2, which influences the proliferation, migration, and apoptosis of endometriosis cells. Nevertheless, the precise mechanism underlying its regulation remains unclear.

### 3.4 Functional Enrichment Analysis and Validation of PNMA2 in Endometriosis

**FIGURE 5:**
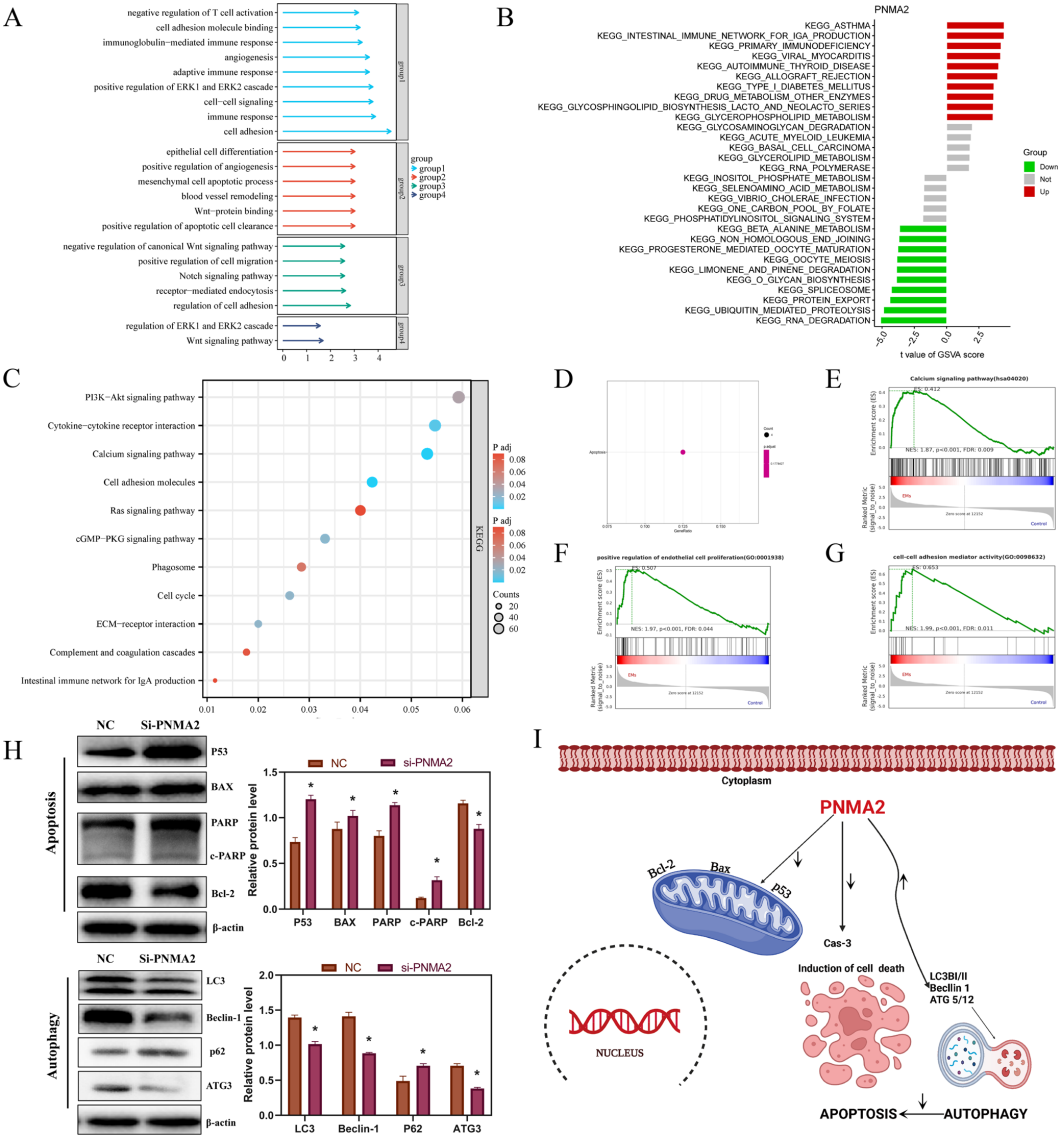
Functional enrichment analysis of PNMA2 in endometriosis. (A) Go (Gene Ontology) functional annotation analysis in endometriosis from RNA‐seq sequencing data of clinical specimens. (B) GSEA pathway enrichment analysis of PNMA2 in endometriosis in the GEO database. (C) KEGG (Kyoto Encyclopedia of Genes and Genomes) pathway enrichment analysis in endometriosis from RNA‐seq sequencing data of clinical specimens. (D) Bubble diagram of pathway enrichment analysis of PNMA2 in endometriosis from the GEO database. (E–G) Results of GSEA enrichment analysis of PNMA2. (H) Western blot results of apoptosis and autophagy signal pathway‐related proteins in PNMA2 knockdown 12z cells. (I) Schematic diagram of the mechanism of apoptosis and autophagy signal pathway regulation in PNMA2 knockdown 12z cells. *p ≤ 0.05.

With the progress of scientific and technological advancements, the utilisation of big data's function enrichment analysis and the predictive capabilities of the signalling axis have facilitated the exploration of new targets in a convenient manner. Consequently, our initial analysis of the PNMA2 gene in endometriosis, utilising data from the GEO dataset, revealed potential functions and signalling axes. Notably, Figure 5B,D indicate a significant association between PNMA2 and immune regulation as well as apoptosis in endometriosis. Transcriptome sequencing of clinical samples revealed the identification of potential functions and regulatory mechanisms that are enriched in endometriosis tissues. Notably, Figure 5A,C indicate the significant involvement of cell adhesion, immune regulation, and phagocytosis in the pathogenesis of this disease. Furthermore, the analysis of an alternative enrichment algorithm conducted by GSEA demonstrated a strong association between the PNMA2 gene and crucial processes such as proliferation, calcium ion regulation, and cell adhesion in endometriosis (Figure 5E–G). Numerous studies have indicated that an excessive accumulation of calcium ions within cellular mitochondria plays a regulatory role in cellular autophagy by either acting synergistically or inhibiting apoptosis.

**FIGURE 6:**
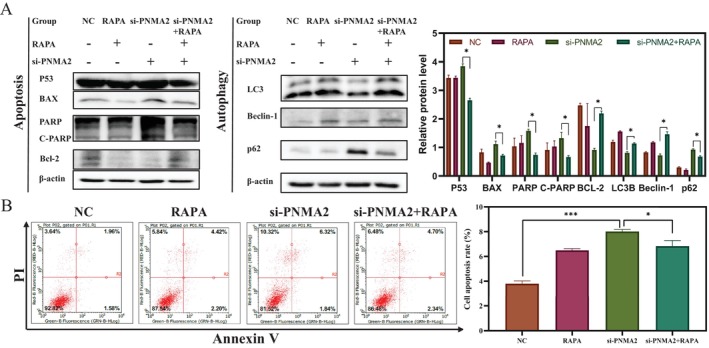
The regulatory mechanism of PNMA2 knockdown on apoptosis and autophagy based on RAPA rescue experiment in endometriosis. (A) Relative expression levels of apoptosis‐ and autophagy‐related proteins in each group based on Western blotting. (B) The percentage of cells in the apoptotic state in each group based on flow cytometry. (A, B) Rescue experiments based on the autophagy activator (RAPA) were performed and divided into four groups, namely NC, RAPA, si‐PNMA2, and si‐PNMA2 + RAPA. *p ≤ 0.05, and ***p ≤ 0.001.

Consequently, in order to investigate its association with apoptosis and autophagy, western blotting experiments were conducted. The Western blotting results in Figure 5H indicated that knocking down PNMA2 (si‐PNMA2) could significantly increase the expression of positive apoptotic proteins (P53, BAX, PARP, c‐PARP and Bcl‐2) and reduce the expression of negative apoptotic proteins (P53, BAX, PARP, c‐PARP and Bcl‐2), which indicated that knocking down PNMA2 could promote cell apoptosis. At the same time, the results in Figure 5H indicated that knocking down PNMA2 (si‐PNMA2) could significantly reduce the expression of positive autophagy proteins (LC3, Beclin‐1 and ATG3) and increase the expression of negative autophagy proteins (P62), which indicated that knocking down PNMA2 could inhibit cellular autophagy by hindering the formation of phagocytic vacuoles and autophagosomes. Based on the above results, it is reasonable to speculate that abnormally high expression of PNMA2 could inhibit apoptosis and promote autophagy for endometriosis. So what is the potential relationship between apoptosis and autophagy affected by PNMA2? Next, this study conducted a rescue experiment based on Rapamycin (RAPA), an autophagy activator, to explore the relationship between PNMA2, apoptosis and autophagy (Figure 6). First, the Western blotting results in Figure 6A suggested that RAPA could effectively promote autophagy inhibited by si‐PNMA2. Then, the flow cytometry results in Figure 6B suggested that RAPA's promotion of autophagy (inhibited by si‐PNMA2) could effectively reduce apoptosis. Based on these results, it is reasonable to speculate that knocking down PNMA2 could directly promote apoptosis, and could also inhibit autophagy and indirectly promote apoptosis. From another perspective, abnormally high expression of PNMA2 could directly inhibit apoptosis, and could also promote autophagy and indirectly inhibit apoptosis. The relevant speculation was shown in Figure 5I.

### 3.5 Immune‐Related Characteristics of PNMA2 in Endometriosis

**FIGURE 7:**
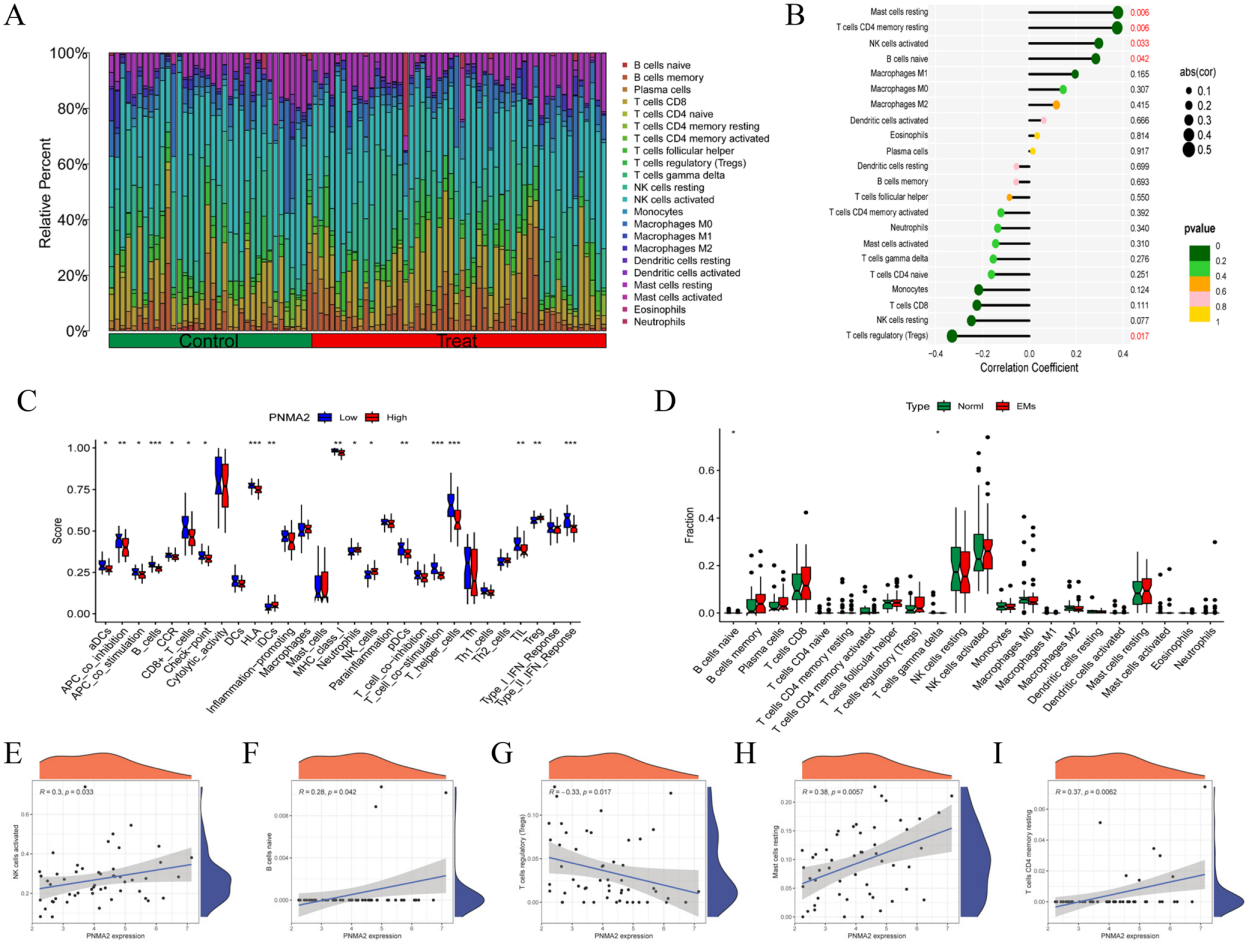
Immune correlation analysis between EMs and normal controls. (A) Heatmap of immune cell correlations in samples. The core heatmap shows the relationship between 22 immune cell abundance ratios. (B) Lollipop plot of PNMA2 gene in relation to the degree of infiltration of 22 immune cells. (C) Statistical violin plot of the difference in the expression of 22 immune cells between the PNMA2 high expression group (red) and the PNMA2 low expression group (blue) (*p* < 0.05). (D) Statistical violin plot of the difference in the expression of 22 immune cells in the EMS group (red) versus the normal control group (green) (*p* < 0.05). (E) Correlation analysis graph of PNMA2 gene expression with NK cells. (F) Correlation analysis graph of PNMA2 gene expression with B cells. (G) Correlation analysis graph of PNMA2 gene expression with Tregs cells. (H) Correlation analysis graph of PNMA2 gene expression with Mast cells. (I) Correlation analysis graph of PNMA2 gene expression with T cells CD4 memory. *p ≤ 0.05, **p ≤ 0.01, and ***p ≤ 0.001.

The enrichment analysis of endometriosis and the PNMA2 gene demonstrated a significant association with immune regulation. The enigmatic and pivotal role of immunomodulation in endometriosis necessitates further elucidation. To enhance our understanding of the diagnostic and therapeutic potential of the PNMA2 gene and its immunomodulatory function, we conducted an analysis of immune cell infiltration. Figure 7A,D depict variations in the proportion and expression of 22 immune cells between the disease group and the normal control group. Correlation analysis of PNMA2 gene with more than 20 types of immune cells was seen in Figure 7B,C. The above analysis suggests that endometriosis has a significant regulatory relationship with T cells, NK cells, B cells, and macrophages. In addition, PNMA2 had an equally significant correlation with T cells, NK cells, and B cells. Figure 7E–I shows that PNMA2 was positively correlated with NK cells (*R* = 0.3), B cells (*R* = 0.28), Mast cells (*R* = 0.38) and T cells (*R* = 0.37). Overall, patients with high PNMA2 expression had multiple immune cell infiltrations, thus suggesting that PNMA2 may serve as a potential immune modulator for the treatment of patients with endometriosis.

## 4 Discussion

Endometriosis is a chronic ailment that imposes a substantial socio‐economic burden. Despite ongoing research, the precise etiological mechanism remains elusive; however, hormonal, genetic, and immunological factors have all been implicated in the pathogenesis of symptoms. Presently, endometriosis faces two significant challenges: suboptimal treatment outcomes and a notable recurrence rate of symptoms [18]. The identification and investigation of novel biomarkers for endometriosis, along with the development of efficacious non‐surgical interventions, offer promising avenues to tackle the aforementioned challenges. This study aimed to investigate the potential role and mechanism of the newly identified endometriosis target PNMA2 through the utilisation of public data, machine learning, clinical sample transcriptome sequencing, and in vitro cell experiments. Additionally, we sought to examine the association between the PNMA2 gene and the immune cell infiltration observed in patients with endometriosis. The results of this study have the potential to enhance our understanding of the immune microenvironment of endometriosis, elucidate that PNMA2 promotes malignant proliferation, migration, and resistance to apoptosis in the disease, and can serve as a potential therapeutic target for endometriosis.

### 4.1 PNMA2 Is Abnormally Overexpressed in Endometriosis During Malignant Progression

In this study, we employed the LASSO and SVM‐RFE dual machine learning algorithms to identify core target genes associated with endometriosis by analysing multiple GEO datasets. The integration of Machine Learning (ML)‐based predictive algorithms has become the preferred approach in biomedical research for uncovering disease‐causing genes, owing to the advancements in high‐throughput genomics platforms [19, 20, 21]. Machine learning techniques have emerged as a valuable tool for effectively predicting outcomes based on extensive and intricate biological data, as well as facilitating the identification and verification of novel clinical targets [22]. Consequently, the key genes associated with machine learning were verified through transcriptome sequencing of patient tissues, ultimately identifying the highly expressed PNMA2 gene in endometriosis diseases at both the mRNA and protein levels. PNMA2 belongs to the paraneoplastic Ma protein family, which exhibits aberrant expression patterns in certain tumour tissues [23]. Furthermore, the promotion of proliferation, invasion, and migration of colorectal cancer cells by PNMA5 is evident [24]. The abnormal expression pattern of PNMA2 indicates its potential significance in tumorigenesis; nevertheless, its involvement in endometriosis remains unexplored [23]. Our investigation has successfully identified PNMA2 as a novel diagnostic biomarker for endometriosis. Consequently, it is imperative to conduct further assessments to elucidate the pathogenic role and mechanism of PNMA2 in endometriosis. This study aimed to investigate the biological functions of PNMA2 from various perspectives, employing cell proliferation assay, migration assay, and apoptosis assay. The findings revealed that the suppression of PNMA2 in 12z cells resulted in reduced cell proliferation and clone formation capacity, indicating that the overexpression of PNMA2 contributed to the malignant proliferation of endometriosis cells. Additionally, the knockdown of PNMA2 led to diminished cell migration ability and an increased count of apoptotic cells. The findings indicate that the upregulation of PNMA2 contributes to the enhanced migratory and anti‐apoptotic capacities of endometriosis cells. Moreover, the precise regulatory mechanism underlying the action of PNMA2 remains elusive, in spite of investigations into its aberrant expression and functional implications.

### 4.2 PNMA2 Inhibits Mitochondrial Apoptosis and Promotes Autophagy

PNMA1 is one of six proteins belonging to the PNMA gene family, which includes PNMA1, PNMA2, PNMA3, PNMA4, PNMA5, and PNMA6A. While PNMA2 has received limited research attention, its inclusion in studies of the gene family suggests a potential regulatory mechanism. The PNMA gene family is known to be involved in cancer and apoptosis signalling. Specifically, PNMA4 has been extensively studied as a regulator of cell death. It facilitates apoptosis by facilitating the interaction between RASSF1A and Bax through the BH3 domain [25]. Additionally, PNMA5 promotes apoptotic signalling in HeLa and MCF‐7 cells, with both its N‐ and C‐terminal domains being essential for its pro‐apoptotic function [26]. Other current research aimed to investigate the potential mechanism of action of the PNMA gene family, known for its involvement in apoptosis regulation. This was achieved through the utilisation of GO and KEGG functional and mechanistic enrichment analyses, employing GEO datasets and transcriptome sequencing data. The findings revealed that PNMA2 exhibited enrichment in cell adhesion, apoptosis, and immune‐related regulation in endometriosis. In order to gain further insight into its regulatory role, the study employed a Western blotting (Figure 5H) to detect changes in the expression of apoptosis signalling axis‐related proteins in 12z cells following PNMA2 knockdown. The data reveal an increase in the expression of P53, BAX, and c‐PARP proteins, while Bcl‐2 expression shows a decrease. As a key molecule in DNA damage repair, the increase of PARP may be related to the DNA damage caused by the knockdown of PNMA2 [27, 28]. In addition, the increase of PARP may also be related to factors such as stress, metabolic regulation, and even interference from experimental techniques, which need to be further explored in subsequent studies. These findings suggest the potential involvement of PNMA2 in the conventional mitochondrial apoptosis pathway. Intriguingly, functional enrichment analysis indicates the potential involvement of PNMA2 in cellular phagocytosis, which aligns with numerous studies highlighting the interconnectedness of cellular autophagy and apoptosis [29]. Then, through Western blotting (Figure 5H), we observed a decrease in the levels of autophagy‐related proteins LC3B and Beclin‐1 upon PNMA2 gene knockdown. These findings suggest that PNMA2 facilitates cellular autophagy to support the anti‐apoptotic properties of cells. Immediately after that, this study conducted a rescue experiment based on RAPA, an autophagy activator, to investigate the relationship between PNMA2, apoptosis, and cellular autophagy (Figure 6). The results showed that the knockdown of PNMA2 could directly promote apoptosis and also inhibit autophagy to indirectly promote apoptosis. Therefore, it could be reasonably inferred that the abnormally high expression of PNMA2 could directly inhibit apoptosis and could also promote autophagy to indirectly synergize with the inhibition of apoptosis.

### 4.3 PNMA2 Is Closely Associated With the Immune Microenvironment of Endometriosis

Despite limited understanding of the pathogenesis of endometriosis, it is widely believed that the immune system plays a pivotal role in its development and pathological processes [15]. This study also identified a noteworthy regulatory association between endometriosis and the infiltration of T‐cells, NK‐cells, B‐cells, and macrophages. The abnormal expression of paraneoplastic MA (PNMA) family members is strongly linked to autoimmune reactions, neurodegenerative disorders, and cancer manifestations, suggesting their potential as targets for autoimmune responses [25]. The present study demonstrates a noteworthy association between changes in PNMA1 expression and the infiltration of immune cells as well as immunomodulatory genes in squamous cell carcinoma of the head and neck [30]. Additionally, the utilisation of the CIBERSORT algorithm revealed a remarkable correlation between PNMA2 and Treg cells, NK cells, and B cells in endometriosis. Notably, the microenvironment of endometriosis, despite being classified as a benign tumour, is a multifaceted biological system that has garnered considerable interest as a dynamic ecosystem. These findings highlight the complex interactions between PNMA2 expression and the immune microenvironment in endometriosis.

### 4.4 Strengths and Limitations

Despite providing valuable insights into the involvement of PNMA2 in endometriosis, our study is subject to certain limitations. Firstly, despite the utilisation of two GEO datasets, the sample size remains relatively small, necessitating a larger sample size to validate our findings. Furthermore, to enhance the applicability of our results, it is imperative to explore the correlation between PNMA2 expression and clinicopathological characteristics, prognosis, and immune cell infiltration in a more extensive and diverse patient cohort. Our research has primarily concentrated on investigating the role of PNMA2, and it is recommended that future investigations explore the intricate interplay between signalling pathways and PNMA2. It is important to note that the potential utilisation of PNMA2 in the context of endometriosis remains speculative, and additional studies are required to ascertain its suitability and effectiveness. Considering its potential as a biomarker, prognostic indicator, and therapeutic target, PNMA2 holds promise for various applications.

## 5 Conclusions

In conclusion, our study presents robust evidence, supported by the integration of public data, machine learning, clinical sample transcriptome sequencing and in vitro cell experiments, indicating a significant up‐regulation of PNMA2 in endometriosis. Moreover, this up‐regulation is strongly correlated with various crucial processes such as proliferation, migration, anti‐apoptosis, autophagy, and immune cell infiltration. Consequently, these findings establish a solid foundation for further investigation into the potential utilisation of PNMA2 as a diagnostic marker for endometriosis. Furthermore, it was revealed that PNMA2 may be involved in shaping the immune environment of this challenging benign tumour. Future research should focus on elucidating the intricate molecular mechanisms through which PNMA2 exerts its influence on the immune response in endometriosis, with the ultimate objective of formulating targeted therapies and immunotherapies for the treatment of this condition.

## Author Contributions

**Mengjun Zhang:** conceptualization (equal), data curation (equal), formal analysis (equal), funding acquisition (lead), writing – original draft (lead). **Zidi Zhang:** validation (equal), writing – original draft (equal). **Jialin Wang:** project administration (equal), resources (equal), writing – original draft (equal). **Haodi Yue:** investigation (equal), methodology (equal), writing – original draft (equal). **Xueling Lou:** software (equal), writing – original draft (equal). **Quanling Feng:** supervision (equal), writing – original draft (equal). **Lindong Zhang:** conceptualization (equal), supervision (equal), visualization (equal), writing – original draft (equal), writing – review and editing (equal).

## Ethics Statement

The study protocol was approved by Third Affiliated Hospital of Zhengzhou University (No. 2023‐098, Zhengzhou, China). The use of patient samples conformed to the declaration of Helsinki. All patients provided informed written consent.

## Consent

All authors of this study agree to the publication of this article.

## Conflicts of Interest

The authors declare no conflicts of interest.

## Supporting information


**Figure S1.** Cell transfection efficiency. (A) Immunoblotting of 12z knockout in endometriotic cells transfected with PNMA2. (B) Immunoblotting graph of endometriotic cell 12z knockout transfected with PNMA2. (C) PCR graph of endometriotic cell 12z knockout transfected with PNMA2.

**Table S1.** The source of antibodies used.

## Data Availability

The data can be obtained through the email under reasonable request: zld8399@163.com.
