# Supplementary figures and images for: Unveiling the Role of PNMA2 in Endometriosis: From Proliferation and Apoptosis to Immunomodulation
Source: J Cell Mol Med. 2025 May 5;29(9):e70576. doi: 10.1111/jcmm.70576 (PMC12051379; doi:10.1111/jcmm.70576)

A

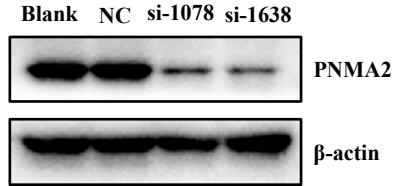

B

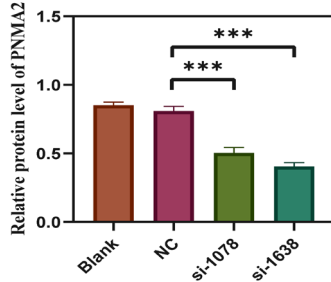

C

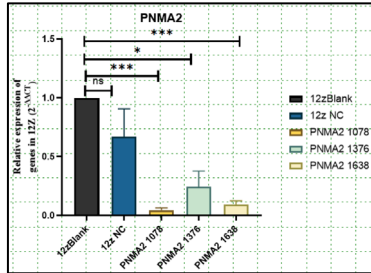

Supplement: Supplementary file 1 — Figure S1. Cell transfection efficiency. (A) Immunoblotting of 12z knockout in endometriotic cells transfected with PNMA2. (B) Immunoblotting graph of endometriotic cell 12z knockout transfected with PNMA2. (C) PCR graph of endometriotic cell 12z knockout transfected with PNMA2. [file JCMM-29-e70576-s002.pdf]
