# Supplementary material for: Unveiling the Role of PNMA2 in Endometriosis: From Proliferation and Apoptosis to Immunomodulation
Source: J Cell Mol Med. 2025 May 5;29(9):e70576. doi: 10.1111/jcmm.70576 (PMC12051379; doi:10.1111/jcmm.70576)
Supplement: Supplementary file 2 — Table S1. The source of antibodies used. [file JCMM-29-e70576-s001.docx]

**Table S1.** **The source of antibodies used.**

| No. | Abbreviation of antibody name | Source | Catalog numbers |
| --- | --- | --- | --- |
| 1 | Beclin-1 | Absin Bioscience Inc., China | abs103411 |
| 2 | LC3-B | Cell Signaling Technology, Inc., USA | 3868 |
| 3 | Bcl-2 | Absin Bioscience Inc., China | abs131701 |
| 4 | Bax | Cell Signaling Technology, Inc., USA | 2772 |
| 5 | β-actin | Affinity Biosciences, China | AF7018 |
| 6 | PARP | Cell Signaling Technology, Inc., USA | 9542 |
| 7 | P53 | Cell Signaling Technology, Inc., USA | 9282 |
| 8 | PNMA2 | Proteintech Group, Inc, China | 16445-1-AP |
